# Promoting sense of coherence: Salutogenesis among people with psoriasis undergoing patient education in climate therapy

**DOI:** 10.1186/2050-7283-1-11

**Published:** 2013-06-21

**Authors:** Eva Langeland, Hilde S Robinson, Torbjørn Moum, Marie H Larsen, Anne-Lene Krogstad, Astrid K Wahl

**Affiliations:** Faculty of Health and Social Sciences, Institute of Nursing, Bergen University College, Møllendalsveien 6, 5009 Bergen, Norway; Medical Faculty, Department of Health Sciences, Institute of Health and Society, University of Oslo, Forskningsveien 3A, 0373 Oslo, Norway; Medical Faculty, Department of Behavioral Sciences in Medicine, Institute of Basic Medical Sciences, University of Oslo, Sognsvannsveien 9, 0372 Oslo, Norway; Department of Rheumatology, Dermatology and Infectious Diseases, Oslo University Hospital, Forskningsveien 1, 0373 Oslo, Norway; Medical Faculty, Institute of Clinical Medicine, University of Oslo, Sognsvannsveien 20, 0372 Oslo, Norway

## Abstract

**Background:**

There is a need for further investigation of sense of coherence (SOC), the central concept of salutogenesis, and its relationship with long-term illnesses such as psoriasis. The aim of this study is to investigate the development of SOC during patient education in the context of climate therapy and to explore factors that may predict changes in SOC among people with psoriasis.

**Methods:**

A prospective design included a baseline assessment and two follow-ups after a 3-week patient education and climate therapy programme (follow-up 1) and again 3 months later (follow-up 2). A total of 254 adults (aged 20–80) with psoriasis participated. SOC was measured by the SOC Questionnaire, illness perception was measured by the Revised Illness Perception Questionnaire, and positive and active engagement in life was measured by the positive and active engagement subscale of the Health Education Impact Questionnaire. Paired-sample *t* tests were used to evaluate changes in SOC from the baseline to follow-up. Multiple linear regression was used to analyse the ability of socio-demographic and clinical variables, illness perception and positive and active engagement in life to predict the changes in SOC.

**Results:**

The SOC score changed significantly by 2.65 points, (95% CI = 0.621, 3.685) from the baseline to follow-up 1. SOC score was still improved by 1.15 points (95% CI = 0.073, 0.223) at follow-up 2.

Baseline and change in positive and active engagement in life were linked to change in SOC with standardized beta 0.170 (95% CI = 0.024, 0.319) and 0.259 (95% CI = 0.092, 0.428), respectively. In addition illness coherence perception at baseline and change in emotional representations were significantly associated with the change in SOC with standardized beta 0.212 (95% CI = 0.073, 0.361) and –0.270 (95% CI = –0.481, –0,077), respectively.

**Conclusions:**

SOC improved significantly from before to after patient education in the context of climate therapy. The results indicate that improving positive and active engagement in life, coping with emotional distress and a coherent understanding of the illness might provide important opportunities to improve SOC among people with psoriasis.

## Background

The concept of sense of coherence (SOC), the main concept in the theory of salutogenesis, may help explain the sustenance for our growth as human beings. Antonovsky (1987) defined SOC as a global orientation expressing the extent to which one has a pervasive, enduring, and dynamic feeling of confidence that the stimuli derived from one’s internal and external environments in the course of living are structured, predictable, and explicable (*comprehensibility*); that the resources are available to meet the demands posed by these stimuli (*manageability*); and that these demands are challenges that motivate one towards investment and engagement (*meaning*). Furthermore, salutogenesis identifies perceived individual and collective general resistance resources (GRRs), such as knowledge, identity, social support, inner feelings, values, coping strategies and different material goods, that may promote the effective management of tension in demanding situations. Higher levels of GRRs are associated with a stronger SOC. The salutogenic approach focuses on peoples’ active adaptation in the interplay between their SOC and use of GRR.

The health of a person could affect their SOC (Veenstra et al. 2005) because it might influence such as the ability to use the different GRR. For instance, a long-term illness such as psoriasis may introduce uncertainty because it is a threat to self, identity, and coping, and may thus challenge the set of life experiences that contribute to a person’s SOC (Antonovsky 1987). It is reasonable to think that the process of active adaptation, including perceptions about illness and engagement in life, shapes life experiences. Awareness of the health care provider about ways to strengthen the patient’s SOC may be important in communication with, and the assessment and treatment of, people with chronic diseases such as psoriasis.

Research focusing only on dysfunction might overlook evidence on human thriving or growth in the context of suffering. Although living with psoriasis can be a source of significant stress and distress (Basavaraj et al. 2011), it is also important to recognize that the disease might not affect the individual only in negative ways. Dealing with everyday adversity arising from psoriasis can bring about substantial growth experiences for patients (Fortune et al. 2005a). It has been suggested that research should examine the patterns and predictors of adversarial growth in psoriasis patients (Fortune et al. 2005b). However, to our knowledge, few studies have investigated salutogenesis including the concept of SOC in individuals with psoriasis. For instance, a study from Poland that included 32 people with psoriasis suggests that SOC may influence how a person manages stress in relation to psoriasis (Oglodek et al. 2009). Schneider et al. (2011) examined 49 psoriasis outpatients and found that feelings of helplessness were a more important contributor to social anxiety and avoidance than were anxious or depressive mood and SOC. In addition, Metz and Jemec (1996) suggested that SOC may provide valuable information about the best ways to help reduce the level of negative psychosocial consequences associated with the skin disease in people with psoriasis.

There is some literature showing that SOC may be strengthened as a result of major life changes or different health care interventions (Langeland et al. 2006;Langeland & Wahl 2009;Yamazaki et al. 2011;Forsberg et al. 2010;Bergman et al. 2011;Habroe et al. 2007). An important focus in the treatment of psoriasis should be on avoiding or reducing the psychosocial consequences of the disease. The treatment should aim to maintain, stimulate, or promote adaptation to illness and positive and active engagement in life. The purpose of this focus is to treat the symptoms and to enable people to live optimally with their psoriasis (Schneider et al. 2011). Climate therapy including patient education is a publicly funded 3-week treatment programme for Nordic countries. Previous studies on such programmes have reported highly significant reductions in disease severity and symptoms (Ben-Amitai & David 2009;Hodak et al. 2003) and improvements in quality of life (Mork & Wahl 2002;Wahl et al. 2005). However, no studies have investigated whether this programme also can improve SOC.

Summed up, little is known about SOC and active adaptation in people with psoriasis, and specifically in relation to changes in SOC following climate therapy. Hence, the main purposes of the present study were to identify changes in SOC in psoriasis patients undergoing patient education in climate therapy and to investigate the ability of positive and active engagement in life versus illness perception to predict changes in SOC. The following research questions were posed.

- Are there associations between socio-demographic and clinical variables and SOC?
- Does SOC change from before to after climate therapy?
- Do the attitudes of positive and active engagement in life and illness perception at baseline predict subsequent changes in SOC?
- Are changes in positive and active engagement in life and illness perception associated to changes in SOC?

## Methods

### Study design and population

The present study was a follow-up study with a pre–post study design of Norwegian patients with psoriasis, age 20 years and older, who were remitted to a climate therapy programme for 3 weeks at the Canary Islands located in the Atlantic Ocean at 28°N, 16°W. The design included three measurements during a period of 4 months. Patients were recruited into the study at the time they were offered climate therapy. Patients answered a questionnaire just before arriving for (baseline) and just before departing after the 3 weeks of therapy (follow-up 1), and again 3 months later (follow-up 2). The study period lasted from late April 2009 to early January 2010. During this time, 343 psoriasis patients were offered climate therapy; 89 declined to participate (reasons not given), giving a total sample of 254 psoriasis patients. For this paper, data were available for 252 (baseline), 248 (follow-up 1), and 205 (follow-up 2) respondents.

### Ethical approval

Approval of the study was obtained from the Norwegian Social Science Data Service and the Regional Committee for Medical Research Ethics for Southern Norway, number 6.2009.440. Participants were coded and analysis was performed anonymously. Procedures were in accordance with the Helsinki Declaration of 1975 as revised in 1983.

### Patient education in the context of climate therapy

The programme comprised both patient education and sun treatment. A Nordic medical team (dermatologist, nurses, and physiotherapist) monitored the patients. Patients received both individual and group-based education, guidance, and daily training. The teaching programme included information and discussions about psoriasis pathogenesis, manifestations, comorbidity, quality of life, and treatment options. The importance of lifestyle choices was stressed with a special focus on physical activity and healthy eating. Discussions in smaller groups focused on finding ways to manage psoriasis in daily life.

In addition patients received on average 80 hours of sun therapy during their stay. The sun exposure was scheduled individually with respect to skin type and UV index. They were encouraged to bathe frequently in salt water and to use moisturizing creams.

### Measures

#### Socio-demographic and clinical variables

Socio-demographic variables such as age, sex, social and marital status, employment status, and educational level were assessed. The following clinical variables were assessed and analysed: years with psoriasis, Psoriasis Area and Severity Index (PASI), comorbidity, and previous climate therapy.

#### Sense of coherence

The Sense of Coherence Questionnaire (SOC-13) was developed by Antonovsky (Antonovsky 1987). This questionnaire is based on self-report and has been tested for validity and reliability in several studies (Antonovsky 1993;Erikson & Lindstrøm 2005;Erikson & Lindstrøm 2006;Feldt et al. 2007;Hittner 2007). In a systematic review it is concluded that the SOC scale seems to be a reliable, valid and cross-culturally applicable instrument measuring how people manage stress and stay well (Erikson & Lindstrøm 2005). SOC-13 comprises 13 items related to comprehensibility (five items), manageability (four items), and meaning (four items). The total score ranges from 13 to 91, with higher scores indicating a stronger SOC. Responses to all items are scored on a seven-point, Likert-type scale. Missing substitution was performed for individuals who answered at least half of the questions for each component. The Cronbach Alpha (α) was 0.829, Intra Class coefficient (ICC) = 0.829 (95% CI = 0.795, 0.859) in the present study.

#### Positive and active engagement in life

The positive and active engagement subscale from the Health Education Impact Questionnaire (heiQ) was used to assess positive and active engagement in life. The subscale comprises five items and is scored using a four-point Likert-type scale from “strongly disagree” to “strongly agree” with higher scores indicating stronger positive and active engagement in life. This construct assesses motivation to be active in health education programmes by engaging or re-engaging in life-fulfilling activities as a result of programme participation. Items in this scale aim to measure the individual’s actions to convert intention into positive outcomes, which imply a change of life activities (Osborne et al. 2007). The HeiQ has high construct validity and is a reliable measure of a broad range of patient education programme benefits (Osborne et al. 2007). The Cronbach α for the positive and active engagement subscale used in the present study was 0.733, ICC = 0.733 (95% CI = 0.677, 0.782).

#### Illness perception

The Revised Illness Perception Questionnaire was used to measure illness perception (Moss-Morris et al. 2002). The questionnaire comprises the following seven components of illness representation: timeline (acute/chronic, six items), consequences (six items), personal control (six items), treatment control (five items), illness coherence (five items), timeline cyclical (four items), and emotional representations (six items). The responses are rated on a five-point Likert scale from “strongly disagree” to “strongly agree”. The Cronbach α for illness coherence, emotional representations and consequences in the present study was 0.870, ICC = 0.870 (95% CI = 0.842, 0.894), 0.856, ICC = 0.856 (95% CI = 0.826, 0.883) and 0.771, ICC = 0.771 (95% CI = 0.723, 0.813), respectively.

### Statistical analysis

The data were analysed using SPSS (version 19.0). Bivariate correlation analyses (Pearson’s r) were used to identify associations between SOC and socio-demographic and clinical variables, and between SOC and illness perception and positive and active engagement in life. Paired *t* tests were used to evaluate the changes in SOC, positive and active engagement in life and illness perceptions from the baseline to follow-up. To investigate the ability of illness perceptions and positive and active engagement in life (both baseline and change) to predict the change in SOC from the baseline to follow-up 1, we used multiple linear regression analysis with the following eligible independent variables after controlling for SOC at the baseline and the socio-demographic and clinical variables: positive and active engagement in life, the illness perceptions of consequences, coherence and emotional representations, respectively. A 5% level of significance was used to include candidate independents.

## Results

### Characteristics of the sample

The mean age of the respondents was 47 years (SD 12, range: 20–80 years). One hundred and two (40%) were women, and 153 (60%) had an educational level of 12 years or less. Seventy (28%) reported living alone. The mean number of years with psoriasis was 24 years (SD = 13, range: 1–60 years). The mean baseline PASI score was 7.5 (SD = 4.1), and 111 (44%) reported comorbidity. Table 1 provides further information about the sample characteristics.

**Table 1:** **Demographic and clinical characteristics of the group at baseline (** ***n*** **= 254)**

		Mean (SD)	Range	n (%)
Sex	Male			152 (60)
	Female			102 (40)
Age		47 (12)	20–80	
Living alone	Yes			70 (28)
	No			184 (72)
Employed	Yes			174 (69)
	No			80 (31)
Education	Primary school: ≤12 years			153 (60)
	University or college: <4 years			52 (21)
	University or college: >4 years			49 (19)
Previous climate therapy	Yes			140 (55)
	No			114 (45)
PASI		7.5 (4.1)	0.4–26.1	
Years with psoriasis		24 (13)	1–60	
Comorbidity	Yes			111 (44)
	No			143 (56)

### Baseline

The mean SOC score at the baseline was 62.8 (SD = 10.3) for the whole sample. SOC correlated significantly with educational level (*r* = 0.156, 95% CI = 0.032, 0.284), age (*r* = 0.134, 95% CI = 0.009, 0.259), duration of psoriasis (*r* = 0.173, 95% CI = 0.046, 0.301), and comorbidity (*r* = 0.176, 95% CI = 0.052, 0.301).

### Relationship between SOC, illness perception, and positive and active engagement in life at baseline

At the baseline, SOC correlated significantly with the following subdimensions of illness perceptions: coherence (*r* = 0.269, 95% CI = 0.148, 0.397), consequences (*r* = –0.369, 95% CI = –0.491, –0.252), and emotional representations (*r* = –0.475, 95% CI = –0.589, –0.360). Furthermore, SOC correlated significantly with positive and active engagement in life (*r* = 0.386, 95% CI = 0.275, 0.515).

### Changes in SOC, illness perception and positive and active engagement in life

The paired-samples *t* test showed a significant change of 2.65 points (95% CI = 1.621, 3.685) in SOC from the baseline to follow-up 1. From the baseline to the follow-up 2, SOC was still increased by 1.15 points (95% CI = 0.073, 2.288).

**Figure 1:**
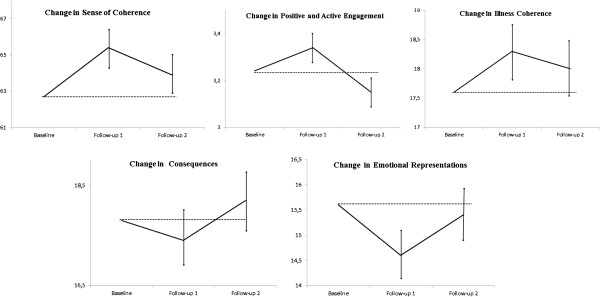
**Baseline and change in key variables with 95% Confidence Intervals for change.**

Changes in illness perception and positive and active engagement in life including mean values with CI 95% for baseline and follow-ups are shown in Table 2 and Figure 1.

**Table 2:** **Mean for the different measures at baseline and follow up 1 and 2**

Measures	Baseline	Follow - up 1	Mean change	95% CI for change	Follow - up 2	Mean change	95% CI for change
	Mean (SD)	Mean (SD)			Mean (SD)		
SOC (13 – 91)^1^	62.8 (10.3)	65.4 (9.9)	2.65	1.621, 3.685	63.9 (10.8)	1.15	0.073, 2.228
Illness coherence (5–25)^1^	17.6 (3.8)	18.3 (3.7)	0.63	0.181, 1.078	18.0 (3.9)	0.32	–0.139, 0.781
Consequences (6–30)^2^	17.8 (4.4)	17.5 (4.6)	–0.38	–0.807, 0.056	18.2 (4.5)	0.39	–0.112, 0.891
Emotional representation (6–30)^2^	15.6 (4.6)	14.6 (4.4)	–0.97	–1.444, –0.491	15.3 (4.4)	–0.24	–0.765, 0.284
Positive and active engagement in life (1–4)^1^	3.24 (.41)	3.34 (.41)	0.11	0.054, –0.159	3.15 (.39)	–0.08	–0.136, –0.025

^1^High = good, ^2^Low = good.

Mean values for the different measures at baseline and follow up 1 and 2 (Figure 1).

### Prediction of change in SOC by baseline and change in positive and active engagement, and illness perceptions

**Figure 2:**
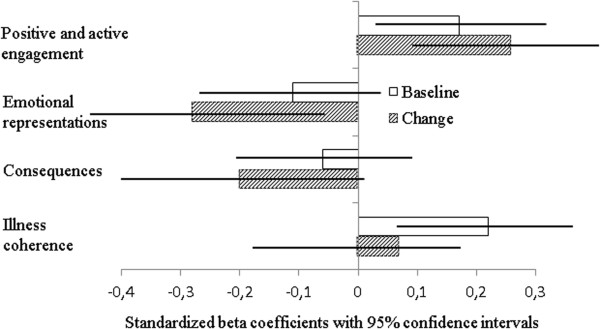
**Relationship between change in Sense of Coherence and baseline and change in independent variables, controlling for sense of coherence at baseline, socio-demographic and clinical variables.**

After controlling for the baseline SOC and the socio-demographic and clinical variables, the multiple regression analysis showed significant associations between both baseline and change in positive and active engagement and the change in SOC score, standardized beta 0.170, (95% CI = 0.024, 0.319) and 0.259, (95% CI = 0.092, 0.428), respectively. While the dimension of illness coherence perception at baseline correlated significantly with the change in SOC score, standardized beta 0.212 (95% CI = 0.073, 0.361) change in emotional representations was significantly related to change in SOC, standardized beta -0.270 (95% CI = –0.481, –0,077). For further information see Figure 2 and Table 3.

**Table 3:** **Relationships between change in SOC and baseline and change in independent variables controlling for baseline SOC and socio-demographic and clinical variables**

	Baseline	Change
Independent variables	Standardized Beta Coefficients(95% CI)	Standardized Beta Coefficients(95% CI)
Positive and active engagement in life^1^	0.170 (0.024, 0.319)	0.259 (0.092, 0.428)
Illness coherence^1^	0.212 (0.073, 0.361)	0.068 (-0.111, 0.251)
Emotional representations^2^	−0.109 (-0.271, 0,046)	−0.270 (-0.481, -0,077)
Consequenses^2^	−0.061(-0.208, 0.085)	−0.092(-0.308, 0.118)

^1^High = good. ^2^Low = good.

Variables associated with change in SOC (Figure 2).

## Discussion

This is the first longitudinal study investigating salutogenesis including SOC in psoriasis patients. We evaluated the ability of factors such as positive attitudes and perceptions related to illness to predict the change in SOC. The present study revealed that SOC improved significantly from before to after climate therapy including patient education in people with psoriasis. We also found that positive and active engagement in life, emotional representations and illness coherence dimension predicted the change in SOC. People with higher levels of positive and active engagement and illness coherence at the baseline had greater improvement in their SOC score following patient education. Further the change in SOC was also significantly associated to change in positive and active engagement and emotional representations.

In the present study, the mean SOC score at the baseline was 62.7 points. This value is lower than that observed in other comparable groups, such as a Swedish general population sample (mean 68.2 points) (Nilsson et al. 2010) and another Norwegian patient group (mean 67 points) (Veenstra & Hofoss 2003), suggesting that there is potential for improving these psoriasis patients’ SOC. Furthermore, SOC was significantly associated with age and education at baseline. Although the correlations in the present study is low, it is in line with earlier research showing that SOC tend to increase with age (Erikson & Lindstrøm 2005) and that education and knowledge are linked to a stronger SOC (Antonovsky 1987). The present study also supports results from other studies showing that SOC is changeable after intervention (Langeland et al. 2006;Yamazaki et al. 2011). (Antonovsky 1979) developed the theory of salutogenesis to counterbalance the emphasis in medicine on pathogenesis, risk factors, and diseases. Antonovsky posed the question “What explains movement towards the health pole of the ease/disease continuum?” (Antonovsky 1996). This is the opposite of the question of which factors create disease. Antonovsky’s answer to his salutogenic question was formulated in terms of the SOC and GRR. Thus, to improve their SOC, people must experience a positive interaction between GRR and SOC. The experience of everyday availability of and interaction with health care personnel, and use of GRR such as new knowledge (education), increased social support, and action competence may contribute to the improvement in SOC observed during patient education and climate therapy. The therapy may provide patients with new experiences of comprehensibility, manageability, and meaning through education and counselling by, and interactions with, health care providers and fellow patients. It is reasonable to think that modification of SOC can occur in settings where health care professionals have long and consistent contact with patients, as is the case with the 3-week patient education and climate therapy programme. However the improvement in SOC was relatively small and decreased again after 3 months. Although the patient education focuses on salutogenic factors such as physical activity, healthy eating and management of psoriasis, it is reasonable to think that with a more explicit salutogenic approach with emphasis on active adaptation in the interplay between SOC and use of GRR, the improvement in SOC could have been stronger and possibly sustained. Nevertheless the relatively small change in SOC may support the hypothesis that SOC is a rather stable quality of an individual after 30 years of age, with only minor fluctuations thereafter (Antonovsky 1987), suggesting that the improvement in SOC in the present study is due to normal variations. However, Antonovsky emphasises that his position is a hypothesis, based on theoretical considerations, and is not based on empirical evidence (Antonovsky 1996). Another hypothesis, based on both theoretical considerations and empirical evidence, is that the strength of SOC may be continuously influenced by external events and internal reactions to these events (Langeland et al. 2006;Langeland & Wahl 2009;Yamazaki et al. 2011;Forsberg et al. 2010). Therefore, more research is required to establish whether SOC can improve in the long term as a result of major changes in life experiences, such as a specific type of therapy with follow-up interventions. The present study indicates the need for follow-up interventions that include a more explicit salutogenic focus, which could promote SOC over the longer term. A methodological limitation of this study is the lack of a control group, which limits any conclusions about cause and effect that may be drawn from this study. Further, 89 patients did not want to participate in the study and we had no opportunity to evaluate the reasons why. Hence, we do not know whether or not this has influenced the results.

People with higher levels of illness coherence at the baseline had greater improvement in SOC following patient education in climate therapy. However in the present study change in illness coherence was not significantly linked to change in SOC so we have to be cautious with our interpretation. Illness coherence assesses the extent to which an individual’s illness provides an opportunity for the patient’s coherent understanding of the illness (Moss-Morris et al. 2002). Bäärnhielm (2005) found that illness coherence relates to SOC through the process of restructuring illness meaning, possibly by constructing coherence between experiences, expression, and past and new meanings given to illness. This process helps strengthen SOC, and may thus be significant for recovery after illness. Nevertheless it is reasonable to think that any change in illness coherence will take time before it will influence the SOC because coherent understanding of the illness and integrate this knowledge may be a longer learning process as suggested by Bäärnhielm (2005). Both baseline and change in positive and active engagement were linked to change in SOC and thus seems to represent an important factor for promoting SOC. This is not a surprising result since this dimension assesses motivation to be active by engaging and reengaging in life–fulfilling activity (Osborne et al. 2007) and is thus an important salutogenic factor. In addition change in emotional representations predicted change in SOC. Emotional representations include negative feelings related to the illness such as depression and anxiety. Earlier research have also revealed that SOC is strongly and negatively associated to emotional distress including anxiety, anger, and depression (Erikson & Lindstrøm 2006) and that SOC is specially related to mental health (Erikson & Lindstrøm 2005).

Altogether illness coherence, positive and active engagement in life and emotional representations, identified as predictors in the present study, may be considered as important components of GRR and thus come into a positive interplay with SOC, by creating good and possibly substantial growth experiences. This is supported by Moss-Morris et al. (2002) showing that illness perception and symptoms can predict different aspects of adaptation to and recovery after chronic disease suggesting that “illness reality” for each individual should play an integral and important role as a collaborative resource alongside other treatments. Further one previous study found a strong positive correlation between reasons for living, such as enjoyment and support in life, and SOC (Yamazaki et al. 2011). Lutz (2009) suggested that SOC may be seen as one’s self-perceived capacity for engagement over time. Participation is a key factor in the theory, and the meaning dimension illuminates this; that is, participation in shaping outcomes and good inner feelings increases SOC (Antonovsky 1987;Sagy & Antonovsky 2000). Thus, patient education in the context of climate therapy that include a focus on positive and active engagement, emotional well-being and illness coherence, may provide the opportunity for the patient to comprehend and integrate the interplay between SOC and such as these GRR.

## Conclusions

SOC improved significantly from before to after patient education in the context of climate therapy. The results indicate that improving positive and active engagement in life, emotional well-being and a coherent understanding of the illness might provide important opportunities to improve SOC among people with psoriasis. The theory of salutogenesis gives a robust description of how to promote coping in the context of SOC. Accordingly, a focus on improving SOC in the treatment of psoriasis may reduce the psychosocial consequences of the disease by improving coping and engagement in life. According to this theory, active adaptation is part of the ideal treatment, which focuses on the adaptive capacity, inner feelings and the dynamics of engagement in life (Antonovsky 1987). This study supports the notion that it is important to focus on emotional well-being, adaptive capacity including illness coherence and the dynamics of engagement in life to promote SOC. Thus, it may be crucial to improve the attitude of positive and active engagement, emotional well-being and illness coherence by enabling patients to use more of their resources and experiences when meeting challenges, thus promoting SOC and health despite the limitations imposed by psoriasis.
